# A 1 × 2 Low-Profile Filtering Antenna Array Using Strip Dense Dielectric Patch

**DOI:** 10.3390/mi14101866

**Published:** 2023-09-29

**Authors:** Yuyan Deng, Mengyu Xu, Shixian Lin, Kai Xu

**Affiliations:** 1 School of Information Science and Technology, Nantong University, Nantong 226019, China; 2 Research Center for Intelligent Information Technology, Nantong University, Nantong 226019, China; 3 Nantong Key Laboratory of Advanced Microwave Technology, Nantong University, Nantong 226019, China

**Keywords:** antenna array, dense dielectric patch, filtering, low-profile

## Abstract

A 1 × 2 low-profile filtering antenna array is proposed, using an H-shape resonator to feed two strip dense dielectric patch (DDP) resonators. The even–odd mode of the H-shape resonator and the TM_δ1_ mode of the strip DDP resonator form the radiation band. Additionally, the odd–odd mode of the H-shape resonator excites the TM_δ2_ mode of the strip DDP resonator, thus achieving an upper-edge radiation null for the filtering response. The H-shape resonator not only participates in the antenna array radiation, but also excites two strip media patches at the same time, avoiding the traditional power distribution network and effectively reducing the complexity of the antenna array. In addition, compared with the reported dielectric filtering antenna designs, this design has the advantageous features of a low profile, a compact structure, wide bandwidth and a simplified structure. For example, the antenna prototype operating at 4.9 GHz achieves 10 dB impedance, a matching bandwidth of 7.1%, a maximum gain of 8.6 dBi and the compact size of 1.29 × 0.18 × 0.038 $\lambda_{0}^{3}$, without requiring a traditional power division network.

## 1. Introduction

In recent years, the filtering antenna array has been widely discussed because it combines both a filtering and radiation function, as well as having the advantages of high integration and low loss of the system. Usually, the filtering antenna array can be realized by combining multiple filtering antennas with a traditional power dividing network but with a large loss. In [1,2,3], a filtering antenna array without a traditional power dividing network was designed with a long microstrip line with a standing wave, balun structure or excitation of an in-phase electric field. However, the above designs had the drawback of a high loss from the metal radiator in high-frequency applications. Recently, the dielectric filtering antenna array has become a hot topic due to its advantages of low loss, small size and lightweight nature. However, the state-of-the-art dielectric filtering designs are all antenna units, and it also has the challenge of having a low profile.

The filtering dielectric resonator antenna (DRA), as a typical dielectric filtering design, has been studied extensively. In [4,5,6], filtering performance is provided for the DRA by cascading the O-shaped bandpass filter [4], the semi-elliptical-shaped bandpass filter [5] and the filtering tapped Marchand balun [6]. In [7,8], the substrate-integrated waveguide cavity is placed under the DRA to form the two-stage filtering DRA. However, both of these have cascaded designs, which have additional loss from the non-radiation structure. In [9], the *H*-field of the DRA HEM_11δ_ mode cancels the *H*-field generated by the loop structure, which produces the filtering DRA but with the larger profile of 0.154 λ_0_. In [10,11,12,13], the DRA are excited by the microstrip line with open stubs [10,11] or by multiple microstrip lines with different lengths [12,13], in order to gain the filtering function. The profile of the antennas was reduced to 0.105 λ_0_. In [14], the differentially coplanar-fed filtering DRA was reported to have a profile of 0.05 λ_0_ but a 10 dB impedance matching bandwidth of only 3.16%. To reduce the profile of DRA, Lai et al. from the City University of Hong Kong developed a dense dielectric patch (DDP) with a microstrip patch as a reference in 2013 [15]. Then, the filtering DDP with a pair of silver-coated slots [16] was investigated. The results showed that the profile could be reduced to 0.026 λ_0_, but the 10 dB impedance matching bandwidth was greatly reduced to 0.7%. It can be seen from [17] that the filtering function can be realized by generating an electric field in the opposite direction between the upper and lower circular DDP, which still has a larger profile.

In this communication, a 1 × 2 filtering strip dense dielectric patch antenna array without a traditional power dividing network is proposed, in order to achieve both a low profile and a wide bandwidth. The electric field distribution of the operating band and the radiation null for the filtering response are introduced. Meanwhile, the influence of the dimensions on the operating modes and radiation null are studied. The design procedure of the proposed antenna is provided to guide the practical design. A performance comparison is given to support the conclusion.

## 2. Antenna Design

**Figure 1:**
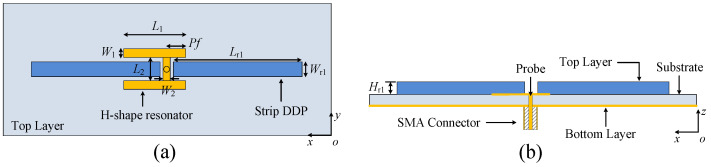
Configuration of the proposed antenna array. (**a**) Top view. (**b**) Side view.

As shown in Figure 1, the proposed 1 × 2 antenna array consists of two strip DDP units, the H-shape resonator at the top layer and a closely placed ground plane at the bottom layer. The two strip DDPs are located on both sides of the H-shape resonator and placed along the symmetrical axis of the H-shape resonator. The substrate is RO4003C substrate (which has a relative permittivity of 3.38, a loss tangent of 0.0027 and the thickness of *h* as 0.813 mm). The antenna array is fed by the probe of a 50 Ω coaxial connector (SMA Connector). The full-wave simulation is achieved by using Computer Simulation Technology (CST).

### 2.1. Strip DDP Resonator

**Figure 2:**
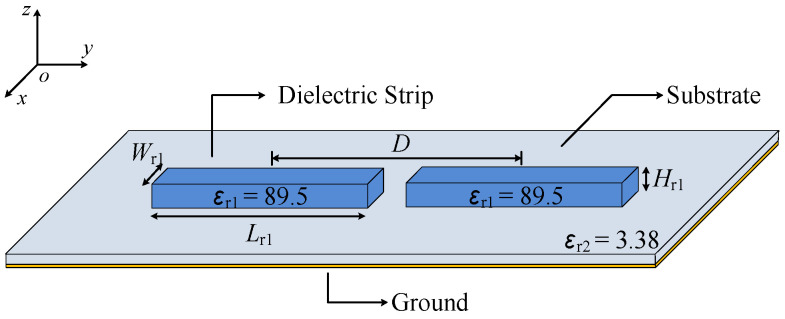
The configuration of two strip DDP resonators.

**Figure 3:**
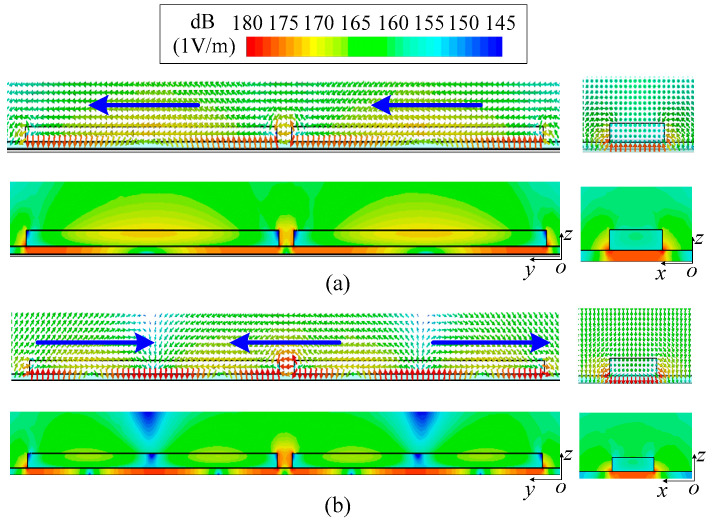
The vector and average magnitude electric field distribution of two strip DDP resonators. (**a**) TM_δ1_ mode. (**b**) TM_δ2_ mode.

Figure 2 shows the configuration of two strip DDP resonators, which consists of two dielectric strips with a high relative permittivity (*ε*_r1_ = 89.5), the substrate with a low relative permittivity (*ε*_r2_ = 3.38) and the ground. The above configuration is used for mode analysis by the Eigenmode solver in CST to explore the method of exciting two strip DDP resonators at the same time. The vector and average magnitude electric field distribution of two strip DDP resonators are exhibited in Figure 3a,b, respectively. In Figure 3a, each strip DDP resonator shows the fundamental TM_δ1_ mode, and there is no electric field phase difference between the two DDP resonators, which can be superimposed in the boresight direction for radiation. In Figure 3b, each strip DDP resonator shows the TM_δ2_ mode, while the electric field phase difference between two strip DDP resonators is 180 degrees, which can be canceled at the boresight direction for the radiation null.

**Figure 4:**
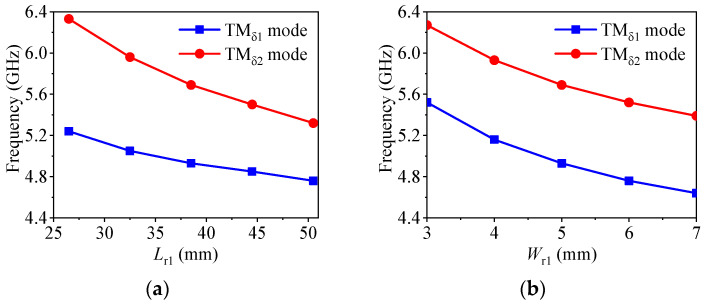

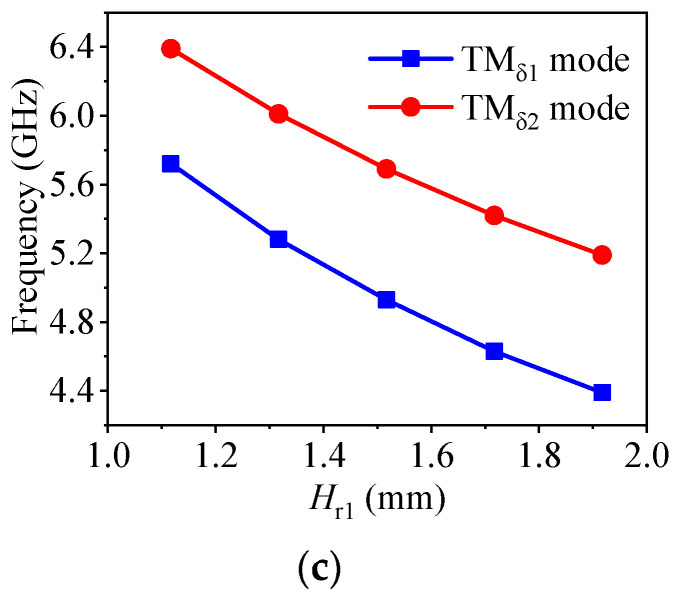
The frequency variations of two modes with different parameters. (**a**) Different *L*_r1_. (**b**) Different *W*_r1_. (**c**) Different *H*_r1_.

Figure 4 exhibits the variation of the resonant frequencies for the TM_δ1_ mode and the TM_δ2_ mode in the strip DDP resonator under different parameters. It can be seen in Figure 4a that, with the increase in *L*_r1_, the resonant frequencies for the TM_δ1_ mode and the TM_δ2_ mode decrease, and the distance between the two modes reduces accordingly. Figure 4b indicates that resonant frequencies of TM_δ1_ and TM_δ2_ modes also show a decreasing trend with an increase in *W*_r1_, and the distance between the two modes is almost unchanged. Finally, as shown in Figure 4c, as *H*_r1_ increases, the resonant frequencies decrease quickly, and the distance between the two modes increases. Therefore, the resonant frequencies for the TM_δ1_ mode and the TM_δ2_ mode in the strip DDP resonator can be controlled by the values of *L*_r1_, *W*_r1_ and *H*_r1_.

### 2.2. H-Shape Resonator

**Figure 5:**
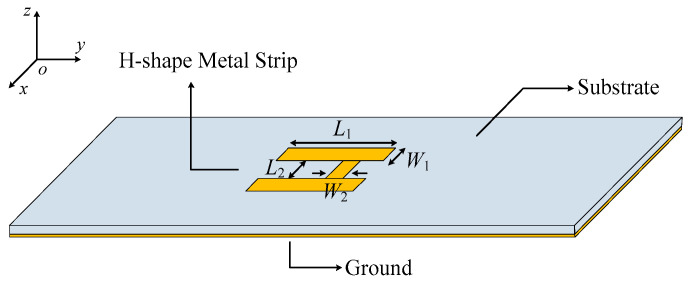
The configuration of the H-shape resonator.

**Figure 6:**
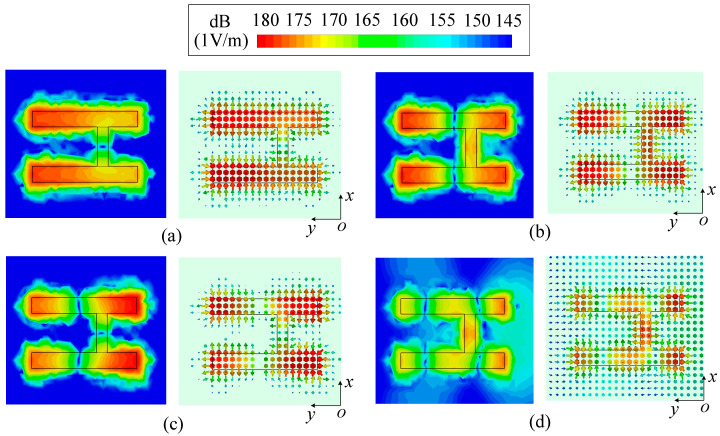
The vector and average magnitude electric field distribution of four modes of the H-shape resonator (*L*_1_ = 16.5 mm, *W*_1_ = 2.5 mm, *L*_2_ = 6.2 mm, *W*_2_ = 1.7 mm). (**a**) Odd–even mode (2.71 GHz). (**b**) Even–odd mode (4.87 GHz). (**c**) Odd–odd mode (5.74 GHz). (**d**) Even–even mode (9.09 GHz).

In order to be able to excite two strip DDP resonators at the same time, an H-shape resonator was designed for power distribution. Figure 5 exhibits the configuration of the H-shape resonator, which consists of an H-shaped metal strip, the substrate and the ground. The four modes of the H-shape resonator in odd- and even-mode analysis are exhibited in Figure 6, which are named as odd–even mode, even–odd mode, odd–odd mode and even–even mode, respectively. It can be seen that the even–odd mode and the odd–odd mode of the H-shape resonator can be used to excite the TM_δ1_ mode and the TM_δ2_ mode of the strip DDP resonator. This is because the electric field distributions at the left and right arms of the even–odd and the odd–even modes are both out-of-phase at different resonant frequencies. Additionally, as the electric field strengths at the left and right arms of the even–odd mode are similar, it can excite the two strip DDP resonators equally.

According to the above analysis, on the one hand, the even–odd mode of the H-shape resonator can be used to excite the TM_δ1_ mode of the strip DDP resonator; thus, the operating radiation band can be formed. On the other hand, the odd–even mode of the H-shape resonator can be used to excite the TM_δ2_ mode of the strip DDP resonator; thus, an upper-edge radiation null can be achieved for the filtering response.

### 2.3. Parametric Study

**Figure 7:**
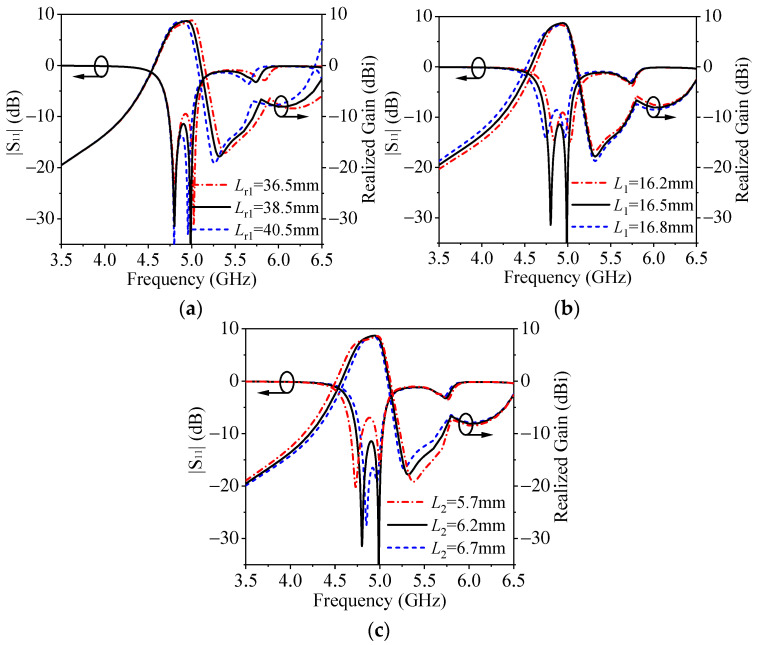
The simulated performance variations under different parameters. (**a**) Different *L*_r1_. (**b**) Different *L*_1_. (**c**) Different *L*_2_.

To further clarify the operating mechanism, Figure 7 shows the effects of *L*_r1_, *L*_1_ and *L*_2_ on the performance of the proposed antenna. It can be seen from Figure 7a that with an increase in *L*_r1_, the second reflection zero and the upper-edge radiation null shift down. This is because the resonant frequencies of the TM_δ1_ mode and the TM_δ2_ mode for the DDP resonator decrease with the increase in *L*_r1_.

Figure 7b indicates that when *L*_1_ increases, the first reflection zero moves down accordingly. The reason is that the even–odd mode of the H-shape resonator decreases with the increase in *L*_1_. Figure 7c shows that with the increase in *L*_2_, the bandwidth decreases and the upper-edge radiation null shifts down. This is because when *L*_2_ increases, the coupling between the H-shape resonator and the DDP resonator decreases, and the resonant frequency of the odd–odd mode of the H-shape resonator decreases.

### 2.4. Design Procedure

According to the above analysis, the design procedure of the proposed antenna is summarized as follows:

(1) Set the operating frequency, bandwidth and radiation zero frequency of the antenna array. Set the center operating frequency of the antenna array to *f*_0_.
(2) By setting *f*_TMδ1_ to *f*_0_, the initial dimensions *L*_r1_, *W*_r1_ and *H*_r1_ of the strip DDP resonator can be obtained from the variation law in Figure 4. The frequency of the even–odd mode for the H-shape resonator needs to be set at *f*_0_ and its initial parameters *L*_1_ and *L*_2_ can be determined from this. The initial parameters *W*_1_ and *W*_2_ can be determined based on the matching of the antenna array.
(3) Tune the dimensions of *L*_r1_, *L*_1_ and *L*_2_ to obtain the final antenna array size according to the parametric study in Figure 7, and then set the operating frequency, bandwidth and radiation zero.

## 3. Results

**Figure 8:**
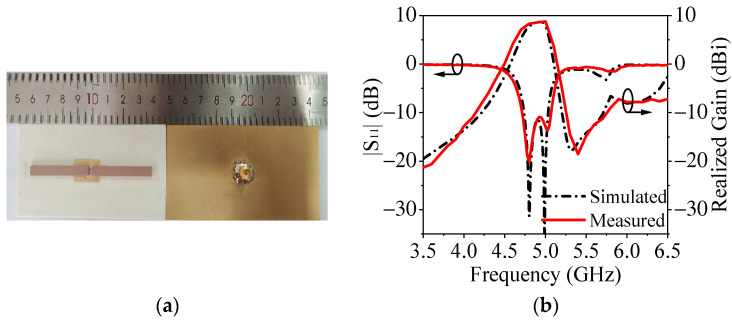
Photograph, simulated and measured |S_11_| and the gain of the proposed antenna. (**a**) Photograph. (**b**) |S_11_| and gain.

To verify the design, a prototype at 4.9 GHz, as shown in Figure 8a, was designed, fabricated and measured. According to the design procedures, the detailed dimensions of the antenna are: *G*_l_ = 98 mm, *G*_w_ = 48 mm, *L*_r1_ = 38.5 mm, *W*_r1_ = 5 mm, *H*_r1_ = 1.5 mm, *L*_1_ = 16.5 mm, *W*_1_ = 2.5 mm, *L*_2_ = 6.2 mm and *W*_2_ = 1.7 mm.

Figure 8b shows the simulated and measured performances of the prototype. The measured 10 dB impedance matching bandwidth is 7.1% (4.73 GHz–5.08 GHz). At the center frequency, the measured gain is about 8.6 dBi. One radiation null occurs at 5.4 GHz.

**Figure 9:**
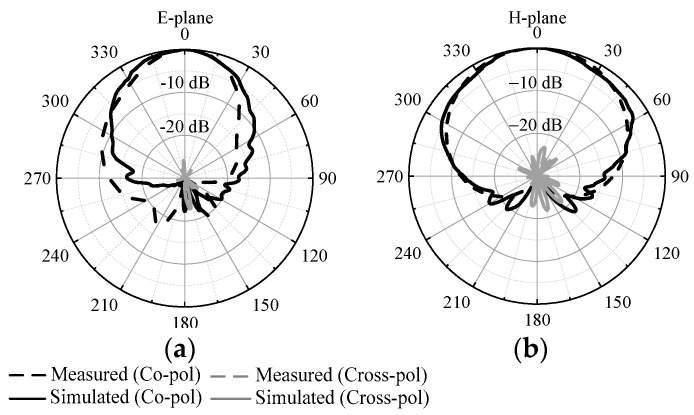
Simulated and measured radiation patterns of proposed design at 4.9 GHz. (**a**) *E*-plane. (**b**) *H*-plane.

Figure 9 exhibits the simulated and measured radiation patterns at 4.9 GHz. The measured 3 dB beamwidth is 46°/90° in the *E*/*H*-plane, the measured front-to-back ratio is above 20 dB in the *E*/*H*-plane and the measured cross polarization level is below −25 dB/−23 dB in the *E*/*H*-plane.

## 4. Discussion

The performances of the proposed design and the reported filtering antenna array are summarized in Table 1. Compared with the dielectric resonant filtering antenna loaded with a notch structure [10,12,13,14], the proposed filtering antenna array design is more compact and has a lower profile. This is because dense dielectric material with a high dielectric constant is used, which can reduce the size of the radiation effectively. Compared with the dielectric patch filtering antenna [16,17], this design achieves both a compact structure and low profile and maintains a wider bandwidth, which is contributed to by the even–odd mode of the H-shape resonator and the TM_δ1_ mode of the strip DDP resonator. In addition, all of the above designs are filtering antenna units, which require a traditional power division network when assembling arrays, and the spacing between the units must be maintained at 0.5–0.7 λ_0_. In this design, the H-shape resonator not only forms the operating band but also plays the role of power distributer, which simplifies the antenna array structure. The main limitation of the proposed design is that the distance between the two strip DDP resonators in the array design is hard to change, as it is limited by the H-shape resonator for power distribution.

## 5. Conclusions

In this communication, a 1 × 2 filtering antenna array is constructed by feeding the strip DDP resonator with an H-shape resonator. The H-shape resonator serves as both a radiator and a power distribution network, which simplifies the antenna array structure. The even–odd mode of the H-shape resonator and the TM_δ1_ mode of the strip DDP resonator are used to form the operating band, which achieves the advantages of a compact structure, a low profile and a wide matching bandwidth. The TM_δ2_ mode of the strip DDP resonator achieves a radiation zero at the high edge of the operating band, which can improve the frequency selectivity and, thus, obtains the filtering response. Compared with the reported dielectric filtering antenna designs, this design has the advantageous features of a compact structure, low profile, wide bandwidth and simplified structure.

## Figures and Tables

**Table 1:** The proposed antenna compared to other related works.

Ref. No	Type	*f*_0_ (GHz)	Profile (*λ*_0_)	Footprint (*λ*_0_ × λ_0_)	Bandwidth (%)	Gain (dBi)	Efficiency (%)	Traditional Power Divider
[10]	DRA	5	0.13	0.67 × 0.43	20.3	9.05	N.A.	Yes
[12]	DRA	1.97	0.12	0.3 × 0.3	21.9	5.1	89.5	Yes
[13]	DRA	2.4	0.105	0.204 × 0.204	7	3.05	N.A.	Yes
[14]	DRA	23.7	0.05	0.67 × 0.47	3.16	7	N.A.	Yes
[16]	DDP	4.17	0.026	0.236 × 0.278	0.7	4.8	N.A.	Yes
[17]	DDP	3.5	0.15	0.327 × 0.327	12.3	9.2	95.9	Yes
This work	DDP	4.9	0.038	1.29 × 0.18 *	7.1	8.6	92.7	No

*: 1 × 2 array.

## Data Availability

The data presented in this study are available on request from the corresponding author.
